# Positive Charges on the Surface of Thaumatin Are Crucial for the Multi-Point Interaction with the Sweet Receptor

**DOI:** 10.3389/fmolb.2018.00010

**Published:** 2018-02-13

**Authors:** Tetsuya Masuda, Satomi Kigo, Mayuko Mitsumoto, Keisuke Ohta, Mamoru Suzuki, Bunzo Mikami, Naofumi Kitabatake, Fumito Tani

**Affiliations:** ^1^Division of Food Science and Biotechnology, Graduate School of Agriculture, Kyoto University, Uji, Japan; ^2^Laboratory of Supramolecular Crystallography, Research Center for State-of-the-Art Functional Protein Analysis, Institute for Protein Research, Osaka University, Suita, Japan; ^3^Division of Applied Life Sciences, Graduate School of Agriculture, Kyoto University, Uji, Japan; ^4^Department of Foods and Human Nutrition, Notre Dame Seishin University, Okayama, Japan

**Keywords:** sweet-tasting protein, positive charge, lysine, electrostatic potential, atomic resolution, SHELXL

## Abstract

Thaumatin, an intensely sweet-tasting protein, elicits sweet taste with a threshold of only 50 nM. Previous studies from our laboratory suggested that the complex model between the T1R2-T1R3 sweet receptor and thaumatin depends critically on the complementarity of electrostatic potentials. In order to further validate this model, we focused on three lysine residues (Lys78, Lys106, and Lys137), which were expected to be part of the interaction sites. Three thaumatin mutants (K78A, K106A, and K137A) were prepared and their threshold values of sweetness were examined. The results showed that the sweetness of K106A was reduced by about three times and those of K78A and K137A were reduced by about five times when compared to wild-type thaumatin. The three-dimensional structures of these mutants were also determined by X-ray crystallographic analyses at atomic resolutions. The overall structures of mutant proteins were similar to that of wild-type but the electrostatic potentials around the mutated sites became more negative. Since the three lysine residues are located in 20–40 Å apart each other on the surface of thaumatin molecule, these results suggest the positive charges on the surface of thaumatin play a crucial role in the interaction with the sweet receptor, and are consistent with a large surface is required for interaction with the sweet receptor, as proposed by the multipoint interaction model named wedge model.

## Introduction

Many low-molecular weight molecules including saccharides, peptides, amino acids, and other synthetic non-nutritive sweeteners (saccharin, acesulfame K, sucralose, cyclamate, and so on), elicit the sensation of sweetness (Nabors and Gelardi, 1991). Most proteins are tasteless and flavorless, but six proteins unexpectedly elicit a sweet taste response on the human palate and are designated as sweet-tasting proteins. They are thaumatin (van der Wel and Loeve, 1972), monellin (Moris and Cagan, 1972; van der Wel, 1972), curculin (neoculin) (Yamashita et al., 1990; Shirasuka et al., 2004; Suzuki et al., 2004), mabinlin (Liu et al., 1993), brazzein (Ming and Hellekant, 1994), and egg white lysozyme (Maehashi and Udaka, 1998; Masuda et al., 2001). Although three-dimensional structures have been determined for thaumatin (Ogata et al., 1992; Ko et al., 1994; Asherie et al., 2009; Masuda et al., 2011a,b, 2014), monellin (Somoza et al., 1993; Lee et al., 1999; Spadaccini et al., 2001; Hobbs et al., 2007), brazzein (Caldwell et al., 1998; Nagata et al., 2013), curculin (Kurimoto et al., 2007), neoculin (Shimizu-Ibuka et al., 2006), mabinlin (Li et al., 2008), and lysozyme (Blake et al., 1967; Phillips, 1967; Smith et al., 1993), no common structural features have yet been found among the sweet-tasting proteins.

Since sweet-tasting proteins are useful as low-calorie sweeteners as well as a substitute for sucrose, a number of investigations have been performed to clarify why sweet-tasting proteins elicit a sweet taste, and how sweet-tasting proteins interact with sweet receptor (Kohmura et al., 1992; Kim and Weickmann, 1994; Slootstra et al., 1995; Somoza et al., 1995; Assadi-Porter et al., 2000, 2010a,b; Kaneko and Kitabatake, 2001; Temussi, 2002, 2006, 2011; Jin et al., 2003; Jiang et al., 2004; Tancredi et al., 2004; Masuda et al., 2005a,b, 2013, 2016; Esposito et al., 2006; Walters and Hellekant, 2006; Koizumi et al., 2007, 2015; Kurimoto et al., 2007; Li et al., 2008; Nakajima et al., 2008, 2011; Ohta et al., 2008, 2011a,b; Ide et al., 2009; Walters et al., 2009; Xue et al., 2009; Dittli et al., 2011; Templeton et al., 2011; Liu et al., 2012, 2016; Cornilescu et al., 2013; Lee et al., 2013; Rega et al., 2015; Ghanavatian et al., 2016; Leone et al., 2016; Lim et al., 2016; Singarapu et al., 2016).

Among the sweet-tasting proteins, thaumatin is ~100,000 times sweeter than sucrose on a molar basis and about 1,600 times sweeter on a weight basis (van der Wel and Loeve, 1972). The threshold value of sweetness of thaumatin to humans is around 50 nM (van der Wel and Loeve, 1972). The intensely sweet taste of thaumatin has been widely used not only as a natural sweetener, but also as a flavor enhancer and for masking unpleasant tastes in the food and pharmaceutical industries (Etheridge, 1994). Identification of the amino residues of thaumatin involved in sweetness should provide important insights into the mechanism of perception of sweet taste and promote the design of novel sweeteners. For these purposes, a number of investigations on thaumatin have been performed, and the basic amino acid residues in the molecule seem to be critical for elicitation of its sweet taste (van der Wel and Bel, 1976; Shamil and Beynon, 1990; Kim and Weickmann, 1994; van der Wel, 1994; Kaneko and Kitabatake, 2001; Ohta et al., 2008, 2011b; Masuda, 2018). Chemical modification studies suggested the importance of lysine residues for elicitation of the sweetness of thaumatin (van der Wel and Bel, 1976; Shamil and Beynon, 1990; van der Wel, 1994). Selective modification showed that five lysine residues (Lys78, Lys97, Lys106, Lys137, and Lys187) located on the same side of the thaumatin molecule play a crucial role in conferring sweetness (Kaneko and Kitabatake, 2001). Site-directed mutagenesis studies revealed that six lysine residues of Lys19, Lys49, Lys67, Lys106, Lys163, and Lys187, and three arginine residues of Arg76, Arg79, and Arg82 located on the cleft-containing side are responsible for sweetness, and two amino acids residues, Lys67 and Arg82, are particularly important for sweetness (Ohta et al., 2008). Mutation studies further revealed that mutations at Arg82 had a more deleterious effect on sweetness than mutations at Lys67, suggesting that a strict spatial charge location appears to be important for the interaction with sweet receptors (Ohta et al., 2011b). More recently, removal of the negatively charged residue Asp21 produced the sweetest thaumatin mutant, D21N, which, with a threshold of only 31 nM with respect to the 50 nM of recombinant thaumatin, is one of the sweetest known molecules (Masuda et al., 2016).

All sweet molecules, such as sugar, saccharides, peptides, amino acids, synthetic non-nutritive sweeteners, and sweet-tasting proteins elicit a sweet taste by interacting the sweet taste receptor T1R2-T1R3, belonging to the family of G-protein-coupled receptors (GPCRs) (Nelson et al., 2001; Li et al., 2002; Xu et al., 2004; Chandrashekar et al., 2006). The sweet taste receptor belongs to class C GPCRs, which comprises metabotropic glutamate receptors (mGluR), γ-amino butyric acid type B receptors, calcium-sensing receptors (CaSR), and many others (Clemmensen et al., 2014). They all possess a large N-terminal extracellular domain, which is composed of two parts, a Venus flytrap (VFT) domain and a cysteine-rich domain. The VFT domain contains the orthosteric active sites that recognize small ligand agonists (i.e., amino acids, calcium, and low molecular sweeteners for mGluR, CaSR, and taste receptors, respectively). However, the interaction of sweet-tasting proteins with the sweet receptor is more difficult to understand with respect to the myriad of low molecular sweeteners, since their dimensions are not consistent with those of the small orthosteric sites in VFT domain of the receptor.

The first attempt to clarify the interaction of sweet-tasting proteins with the sweet receptor was made by Temussi (2002). The peculiar mode of interaction, which is now known as “wedge model” (Tancredi et al., 2004), was based on the equilibrium of the homologous mGluR1 glutamate receptor between two conformations. One is a resting form, in which the two extracellular domains are both open, and the other is an active form, in which one of the two extracellular domains is closed (Kunishima et al., 2000). On the basis of the sequence similarity between the mGluR1 and the T1R2-T1R3 sweet receptor, it was possible to build a reliable homology model of the sweet receptor. The position of the equilibrium between the active and resting forms is shifted by binding low molecular sweeteners in the orthosteric binding sites of VFT domain but also by complexing sweet-tasting proteins on a large external surface (Temussi, 2002; Morini et al., 2005). As a “proof of concept,” Temussi showed by docking calculations that indeed the three paradigmatic sweet-tasting proteins monellin, brazzein, and thaumatin can bind on the outer surface of the active form of the sweet receptor, thus shifting the equilibrium (Temussi, 2002). More recently, Temussi studied the feasibility of building quantitative models of the complexes of sweet-tasting proteins with the sweet receptor on the basis of the wedge model and showed that, among the many fuzzy models of low-resolution docking, it is possible to find topological solutions consistent with mutagenesis data (Temussi, 2011). The complex of monellin found by this approach is fully consistent with a previously reported supersweet MNEI (Esposito et al., 2006). As to thaumatin, a hyper-sweet thaumatin was obtained by the mutation at D21N (Masuda et al., 2016). To explain the surprising increase of sweetening power induced by this seemingly simple mutation, we recurred to the best available procedure, i.e., the tethered docking in the framework of the wedge model (Temussi, 2002, 2011, and references therein). Our thaumatin-sweet receptor complex model confirmed that each of the positively charged residues, including two critical residues (Lys67 and Arg82) is adjacent to a receptor residue of opposite charge to yield favorable electrostatic interactions. Besides these two critical residues, several amino acids residues seem to have the potential to participate in interaction with sweet receptors. These include lysine residues Lys78, Lys106, and Lys137. In order to validate this complex model further, the charge distributions as well as electrostatic potentials of three lysine residues (Lys78, Lys106, and Lys137), which were expected to locate on the interaction sites, should be re-examined through the effects of mutations in accordance with sensory analysis. Particularly, it should be important to check whether or not unexpected conformational changes and the subtle structural changes were induced by mutation, since the subtle conformational changes at the position of 82 resulted in a significant reduction of sweetness (Ohta et al., 2008).

Among the three lysine residues (Lys78, Lys106, and Lys137), Lys106 seems to be unique because it is located in the cavity of the cleft side of the thaumatin molecule. Based on previous chemical modification results, the threshold values of chemically modified phosphopyridoxylated (PLP)-Lys106 and dephosphorylated-PLP-Lys106 are around 300 nM, suggesting Lys106 itself plays a critical role in sweetness (Kaneko and Kitabatake, 2001). In contrast, the threshold value of sweetness of mutant K106A was 140 nM (Ohta et al., 2008). This discrepancy might be partly due to the presence of the relatively bulky PLP molecule and the change of charge distributions at the position of the ε-amino group of Lys106 (Masuda, 2018). Since the structure and the charge distribution on the surface of the thaumatin molecule play a significant role in sweetness, comparison of the structures of mutants with that of wild-type thaumatin at the atomic level would provide important insights into the electrostatic potentials and charge distribution on the surface of thaumatin involved in the interaction with sweet receptor and would clarify subtle structural changes and differences induced by mutation.

In the present study, three mutant X-ray crystallographic structures of thaumatin (K78A, K106A, and K137A) were determined at resolutions of 1.07, 1.07 and 1.01 Å, respectively. The similarities and differences between the refined structures were investigated at the atomic level. The features of electrostatic potentials on the surface molecules should provide important insights into how thaumatin interacts with sweet receptor.

## Materials and methods

### Materials

The specially prepared reagent N-(2-acetamido) of iminodiacetic acid (ADA) was from Dojindo Laboratories (Kumamoto, Japan). Sodium potassium tartrate was obtained from Wako Pure Chemical Industries Ltd. (Osaka, Japan). All other chemicals were of guaranteed reagent grade for biochemical use.

### Expression and purification of thaumatin mutants

A vector carrying a mature thaumatin I gene with a pre-sequence of thaumatin was used as the template (Ide et al., 2007). Site-directed mutagenesis was performed using the QuikChange site-directed mutagenesis kit (Stratagene, La Jolla, CA, USA) as described previously (Ohta et al., 2008). The vector containing the desired mutation (K78A, K106A, or K137A) was digested by *Xba* I and *Csp45* I and the resulting digested fragments ligated into the yeast shuttle vector pPIC6. The pPIC6 vector carrying the desired mutations was linearized using *Pme* I and transformed into the *Pichia* X-33 strain by electroporation (Electroporator 2510, Eppendorf, Hinz GmbH, Hamburg, Germany). A 7-L fermenter (TS-M7L, Takasugi Seisakusho Co., Tokyo, Japan) with temperature and dissolved oxygen controlled units was used for the secretion of mutant protein into the medium (Ohta et al., 2008; Masuda et al., 2010). After centrifugation at 5,000 × g, the culture supernatant was extensively dialyzed against 5 mM sodium phosphate buffer, pH 7.0, containing 0.5 mM EDTA. The dialysate was applied on an SP-Sephadex cation exchange column (GE Healthcare Bio-Science AB, Uppsala, Sweden) and eluted with 5 mM sodium phosphate buffer, pH 7.0, containing 0.5 M NaCl. The fractions containing thaumatin derivative were collected and precipitated by ammonium sulfate. The precipitate was collected by centrifugation at 8,000 × g for 30 min, and further purified by HW-50F gel-filtration chromatography (Tosoh Co., Tokyo, Japan) as described previously (Ohta et al., 2008; Masuda et al., 2010). The purity of proteins was checked by SDS-PAGE with 13.5% polyacrylamide gel as described before (Ohta et al., 2008). The protein concentration of plant thaumatin was determined spectrophotometrically based on the ${\text{A}}_{1\text{cm}}^{1\%}$ at 278 nm = 7.69 (van der Wel and Loeve, 1972). The concentration of mutant protein was determined using the bicinchoninic acid protein assay (Thermo Scientific, Rockford, IL, USA) with plant thaumatin as a standard.

### Sensory analysis of thaumatin mutants

The sweetness threshold of the samples was evaluated by means of a triangle test in humans for taste threshold (Kaneko and Kitabatake, 2001; Masuda et al., 2005b). Five or six subjects (aged 22–38 years) participated in this trial. The threshold value of all participants against intact thaumatin ranged from 40 to 60 nM, and their average is 50 nM. All subjects gave their informed consent in our sensory analysis and written informed consent and agreement was obtained from the participants. The protocol of which was approved by the Graduate School of Agriculture, Kyoto University. The threshold value of sweetness of K106A was previously determined to be a 140 nM and in this study we attempted to determine the threshold values of sweetness for K78A and K137A. Three paper cups, one containing 5 mL of protein solution and the others containing 5 mL of distilled water, were prepared. Sweetness intensity was evaluated on a scale of 0–5 using a scaling bar. The value 0 means no taste detected from the test solution; 1 means that the sample solution elicited some taste stimulation; and 2 represents the concentration at which the panel member detected sweetness from the sample solution. That is, the threshold value of sweetness is the concentration giving a value of 2. The sample solutions were provided in order of concentration from the lowest (50 nM) to the highest (300 nM) in increments of 50 nM. The threshold values were averaged and analyzed with one-way analysis of variance. A post hoc test was performed by Bonferroni and Scheffe test. A *P* < 0.05 was considered as a significant difference in the statistical analysis.

### Crystallization

The purified thaumatin mutants were concentrated by using VIVACON 2 (Sartorius Stedim Biotech GmbH, Goettingren, Germany), and the protein concentration was measured with a NanoDrop ND-1000 spectrophotometer (NanoDrop Technologies, Inc., Rockland, DE, USA). Crystallization was performed using the hanging-drop vapor-diffusion method. The hanging drops were prepared by mixing 5 μL of 30–50 mg/mL protein solution with 5 μL reservoir solution (0.1 M ADA, 0.75 M potassium sodium tartrate, pH 7.0).

### Structural refinement and validation

Diffraction data on mutant thaumatin (K78A, K106A, and K137A) were collected using a RAXIS-V area detector (Rigaku, Tokyo, Japan) at the BL26B1 station of SPring-8 (Hyogo, Japan) (Ueno et al., 2006). The data obtained were processed, merged, and scaled using the HKL2000 program package (Otwinowski and Minor, 1997). Initial rigid body refinement using the structure of thaumatin I (1.10 Å, PDB entry 3al7) as a reference (Masuda et al., 2011a) was performed at a resolution of 1.5 Å using the SHELXL97 and SHELXL-2013 programs (Sheldrick and Schneider, 1997; Gruene et al., 2013). The models were rebuilt using the Coot program (Emsley and Cowtan, 2004) and subsequent refinements were performed using SHELXL. Water molecules were incorporated where the |Fo|-|Fc| electron density map showed peaks above 3σ and density above 1σ was present for the 2|Fo|-|Fc| map. All reflections were included with no σ cut-off; 5% of the data were randomly selected and omitted during refinement for cross validation by means of the free *R*-factor (Brünger, 1992). The occupancies of the alternative conformations were treated as free variables and refined using the FVAR restraints of SHELXL. Anisotropic *B*-factor refinement was performed using the SHELXL package, and finally hydrogen atoms were generated based on the HFIX command of SHELXL. Hydrogen atoms were included only in the protein atoms but not in tartrate/glycerol/solvent atoms. Data collection and structure solution statistics are shown in Table 1. In order to estimate the standard deviations (esds), an unrestrained refinement was attempted by setting the shift multiplication parameters to zero (Cruickshank, 1999). To assess the precision of the obtained structures, the esds of the structure were plotted against thermal parameters by SHELXPRO. The carbon, nitrogen, and oxygen atoms relatively converged to low esd values (Supplementary Figure 2). The quality of the final model was assessed using PROCHECK (Laskowski et al., 1993) and RAMPAGE (Lovell et al., 2003). The CCP4 package was used for the manipulation of data and coordinates (Winn et al., 2011). The electron density maps and structural images were generated using PyMOL (DeLano, 2002). The analysis of Poisson-Boltzmann electrostatics calculations was performed using PDB2PQR Server (Dolinsky et al., 2004). The coordinates and observed intensities have been deposited in the PDBj (PDB ID, 5YYQ for K78A, and 5YYR for K106A, and 5YYP for K137A).

**Table 1:** Data collection and refinement statistics.

	**K78A**	**K106A**	**K137A**
Data collection			
Beamline	SPring-8 BL26B1	SPring-8 BL26B1	SPring-8 BL26B1
Detector	RIGAKU R-AXISV	RIGAKU R-AXISV	RIGAKU R-AXISV
Space group	*P*4_1_2_1_2	*P*4_1_2_1_2	*P*4_1_2_1_2
Cell dimension (Å)	a = b = 57.719, c = 150.386	a = b = 57.639, c = 149.939	a = b = 57.742, c = 149.986
X-ray wavelength (Å)	0.80	0.80	0.80
Resolution limit (Å)	50.00–1.07 (1.09–1.07)^†^	50.00–1.07 (1.09–1.07)^†^	50.00–1.01 (1.03–1.01)^†^
Unique reflections	112,490	110,727	133,031
Redundancy	10.7 (10.6)	9.9 (7.1)	6.6 (6.6)
Completeness (%)	99.9 (99.8)	98.9 (93.9)	99.9 (100)
<I/σ(I)>	71.26 (8.20)	60.68 (5.54)	44.07 (5.94)
*R*_merge_	0.056 (0.496)	0.067 (0.497)	0.064 (0.480)
*R*_meas_	0.059 (0.521)	0.070 (0.534)	0.069 (0.521)
CC_1/2_	0.999 (0.964)	0.999 (0.899)	0.999 (0.924)
Refinement	SHELXL	SHELXL	SHELXL
Resolution (Å)	20.00–1.07	20.00–1.07	20.00–1.01
Unique reflections	112,385	110,509	132,932
*R*_work_/*R*_free_ (*F*o > 4σ)	13.3/14.1 (11.1/12.6)	12.1/14.0 (11.4/13.2)	11.7/13.6 (10.8/12.5)
*R*_cryst_ (*F*o > 4σ)	11.7 (11.1)	12.1 (11.4)	11.8 (10.8)
Protein atoms	3,284	3,267	3,309
Tartrate atoms	20	20	10
Glycerol atoms	24	18	18
Solvent atoms	279	300	363
Average B factor (Å^2^)			
Whole chain	17.37	16.09	13.14
Protein main chain	12.06	11.07	8.94
Protein side chain	19.39	17.98	14.72
Tartrate	16.38	17.82	6.90
Glycerol	38.16	33.05	27.74
H_2_O	33.46	30.83	26.50
R.m.s.d. bond (Å)	0.017	0.017	0.017
R.m.s.d. angle (Å)	0.035	0.034	0.037
PDB ID	5YYQ	5YYR	5YYP

† *Values in parenthesis are for the highest resolution shell*.

### Models of complexes between proteins and sweet taste receptor

Models of the complexes between thaumatin mutants and the sweet taste receptor were obtained as previously described (Temussi, 2011). Briefly, the ensembles of complexes of thaumatin (PDB entry 3al7, Masuda et al., 2011a) with the T1R2-T1R3 sweet receptor were built using the GRAMM software in a low resolution mode (Vasker et al., 1999). The thaumatin-receptor complexes were refined using GRAMM-X (http://vakser.bioinformatics.ku.edu). In GRAMM-X, it is possible to add receptor residues hypothesized to be at the interface of the complex from the low resolution mode and thaumatin residues suggested by mutagenesis data. We favored charged residues because of the importance of electrostatic interactions in the model. The following residues were selected for receptor (T1R2: D169, E170, R172, D173, K174, R176, D213, R217, D218, D456, R457), and (T1R3: R177, D190, R191, D216). And we selected K49, K67, K97, K106, K137, K163, K187, R76, R79, and R82 for thaumatin. The other parameters were maximized as described (Temussi, 2011).

## Results and discussion

### The effects of mutation of lysine residue on thaumatin sweetness

The results of the sensory analysis of K78A and K137A are shown in Table 2. The threshold values of K78A and K137A were 220 and 230 nM, respectively, and sweetness decreased by ~5-fold when compared to recombinant and plant thaumatin. Although 11 lysine residues are present in a molecule, the effects of substitution of lysine residue with alanine residue on sweetness were examined for 7 lysine residues (Lys19, Lys49, Lys67, Lys78, Lys106, Lys137, and Lys163). The threshold value of K67A is 870 nM, and sweetness decreased by ~19-fold, suggesting that Lys67 is a critical residue for sweetness. The threshold values of sweetness of K49A, K106A, and K163A were 140, 140, and 180 nM, respectively, which is about a 3.1- to 4-fold reduction of sweetness. Chemical modification studies suggested that Lys106, in particular, seems to be important for sweetness (Kaneko and Kitabatake, 2001). The results of site-directed mutagenesis of K106A (threshold value is 140 nM) differed from those of chemical modification, in which the threshold values of PLP-K106 and dephosphorylated-PLP-K106 were around 300 nM. The discrepancy between two studies might be partly due to the rather obvious fact that the relatively bulky PLP molecule would interfere with other adjacent amino acid residues and might disrupt conformations nearby. In fact, the distance between Lys106 and Lys49 is about 9 Å, and those of Lys106 and Arg82 are 10–11 Å, suggesting that bulky chemically-modified derivatives at the position of Lys106 might affect the interaction sites of thaumatin, particularly against Arg82, causing an unexpected reduction of sweetness (Masuda, 2018). The results of R82K might support and strengthen this conclusion, since the subtle conformational change at position 82 led to a remarkable reduction of sweetness. Thus, it seems that detailed structural determination at an atomic level by X-ray structural analysis should help understand whether local conformational changes were induced by mutation at adjacent interaction sites.

**Table 2:** Effects of Mutation of Lys residues on sweetness of thaumatin.

	**Sweetness threshold (nM)**
K78A^§^	223 ± 36
K137A^§^	230 ± 36
plant^§^	50 ± 3
K106A^¶^	140 ± 19
K19A^¶^	70 ± 0
K49A^¶^	140 ± 10
K163A^¶^	180 ± 24
K67A^¶^	870 ± 92
plant^¶^	45 ± 9
Recombinant^¶^	45 ± 12

*Mean ± SE*,

§ *Data obtained in this study*.

¶ *Data from Ohta et al. (2008)*.

### Structure of K78A thaumatin

**Figure 1:**
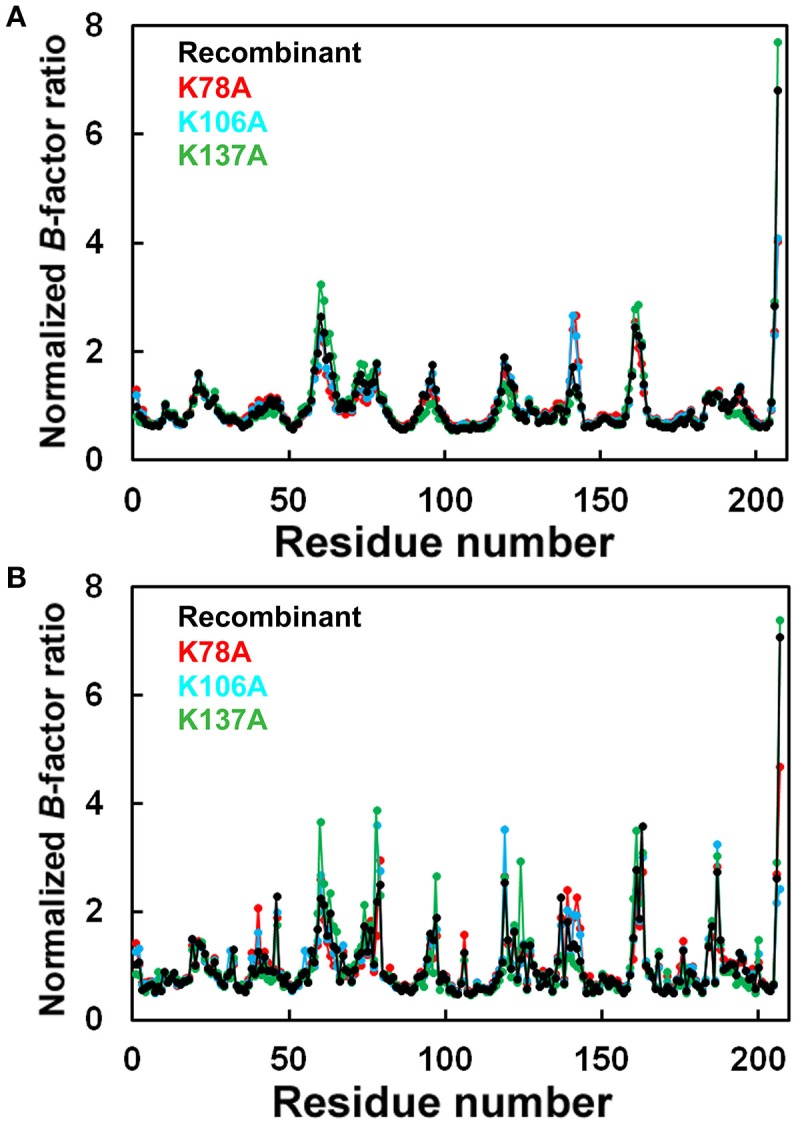
Comparison of normalized *B*-factor values among thaumatin mutants. Histograms of Normalized *B*-factor (*B*-factor of each residue/*B*-factor whole average) with the residue number. **(A)** Main-chain for K78A (red), K106A (blue), K137A (green), and recombinant thaumatin (black). **(B)** side-chain for K78A (red), K106A (blue), K137A (green), and recombinant thaumatin (black) thaumatin. The structural data of recombinant thaumatin was from PDB entry 3al7 (Masuda et al., 2011a).

The final model of the structure of K78A thaumatin consists of a total of 3,284 protein atoms including hydrogen atoms, 20 tartrate atoms, 24 glycerol atoms and 279 water molecules. The average *B*-factors for the main-chain and side-chain were 12.06 and 19.39 Å^2^, respectively (Table 1). Anisotropic *B*-factor refinement resulted in the *R*_*work*_ and *R*_*free*_ factors converging to 13.3 and 14.1%, respectively. Final refinement with riding hydrogen atoms and including all reflections resulted in the *R*_*cryst*_ factor converging to 11.7%. The *B*-factors of the main-chain and side-chain of mutated residue Ala78 were 19.64 and 28.49 Å^2^, respectively. The normalized *B*-factor ratio (*B*-factor of residue/Average *B*-factor) of this residue was 1.63 for main-chain and 1.55 for side-chain (Figure 1). These values were substantially lower than the normalized *B*-factor ratio of Lys78 of recombinant thaumatin (1.77 for main-chain and 2.20 for side-chain) in which the *B*-factors of main-chain and side-chain are 17.44 and 31.24 Å^2^, respectively. The normalized *B*-factor ratio was reduced 0.65 for side-chain, suggesting that the flexibility of the position of the mutated site of 78 seemed to decrease. Superposing the structure of K78A on that of recombinant thaumatin (PDB: 3al7) showed no obvious differences in r.m.s.d. values except alternative side-chains and the C-terminal residue of Ala207, suggesting that the overall structure of K78A is similar to that of recombinant thaumatin (Supplementary Figure 1). These results suggested that no significant structural changes were induced by the mutation from the comparisons on the structures at atomic resolution, and that the flexible conformation of Lys78 side-chain might help to optimize the interaction with the opposite charged residue of sweet receptor.

### Structure of K106A thaumatin

The *R*_*work*_ and *R*_*free*_ factors for the final structure of K106A thaumatin were 12.1 and 14.0%, respectively. The structure consists of 3,267 protein atoms including hydrogen atoms, 20 tartrate atoms, 18 glycerol atoms and 300 water molecules. The average *B*-factors for the main-chain and side-chain were 11.07 and 17.98 Å^2^, respectively (Table 1). Positional uncertainties against thermal parameters were also calculated as with K78A and a similar result was obtained to K106A (Supplementary Figure 2). The *B*-factors of main-chain and side-chain of mutated residues of Ala106 were 7.55 and 12.27 Å^2^, respectively. In Lys106, the *B*-factors of main-chain and side-chain were 5.94 and 17.81 Å^2^, respectively. The normalized *B*-factor ratio of Ala106 was 0.68 for main-chain and 0.72 for side-chain and those of Lys106 in recombinant thaumatin for main-chain and side-chain were 0.61 and 1.25, respectively (Figure 1). The normalized *B*-factor ratio decreased by 0.53 for side-chain, suggesting that the flexibility of side-chain was also reduced. Compared to the K78A, moderate reduction of flexibility seems to be induced by the mutation of K106A. Besides, the flexibility of Lys106 is substantially lower than that of Lys78 and Lys137 in wild type structure, suggesting that the lower flexibility of side-chain at interaction sites might not help support good complementarity with sweet receptor. The results of superposition between K106A and recombinant thaumatin showed no obvious differences in r.m.s.d. values except alternative side-chains and Ala207 as with K78A (Supplementary Figure 1). The detailed structural assignment at an atomic level revealed no significant conformational changes in interaction sites of Ala106 as well as Arg82 were induced by mutation.

### Structure of K137A thaumatin

The final model of K137A thaumatin was determined at a slightly higher resolution of 1.01 Å. It consists of 3,309 protein atoms, including hydrogen atoms, 10 tartrate atoms, 18 glycerol atoms and 363 water molecules. Compared to K78A, K106A, and recombinant thaumatin, one tartrate molecule was reduced in K137A. The average *B*-factors for the main-chain and side-chain were 8.94 and 14.72 Å^2^, respectively (Table 1). These values were lower than those of K78A and K106A. The accuracy of the model for K137A was shown by positional uncertainties against thermal parameters as with K78A and K106A. The *B*-factors of main-chain and side-chain of mutated residues of Ala137 were 8.87 and 16.35 Å^2^, respectively. In Lys137 of recombinant thaumatin, the *B*-factors of the main-chain and side-chain were 9.33 and 32.24 Å^2^, respectively. The normalized *B*-factor ratios of Ala137 were 1.00 for main-chain and 1.18 for side-chain, and those of Lys137 in recombinant thaumatin for main-chain and side-chain were 0.95 and 2.27, respectively. The normalized *B*-factor ratio decreased by 1.09 for side-chain, suggesting that the flexibility of the side-chain of Lys137 was substantially reduced (Figure 1). The reduction of flexibility at the interaction sites indeed seems to be consistent with the difference in sweetness, since the threshold value of K137A (230 nM) and K78A (220 nM) is higher than that of K106A (140 nM). The superposition between K137A and recombinant thaumatin showed no obvious differences in r.m.s.d. values except alternative side-chains and C-terminal Ala207 as with K78A and K106A (Supplementary Figure 1).

### Comparison of electrostatic potentials

**Figure 2:**
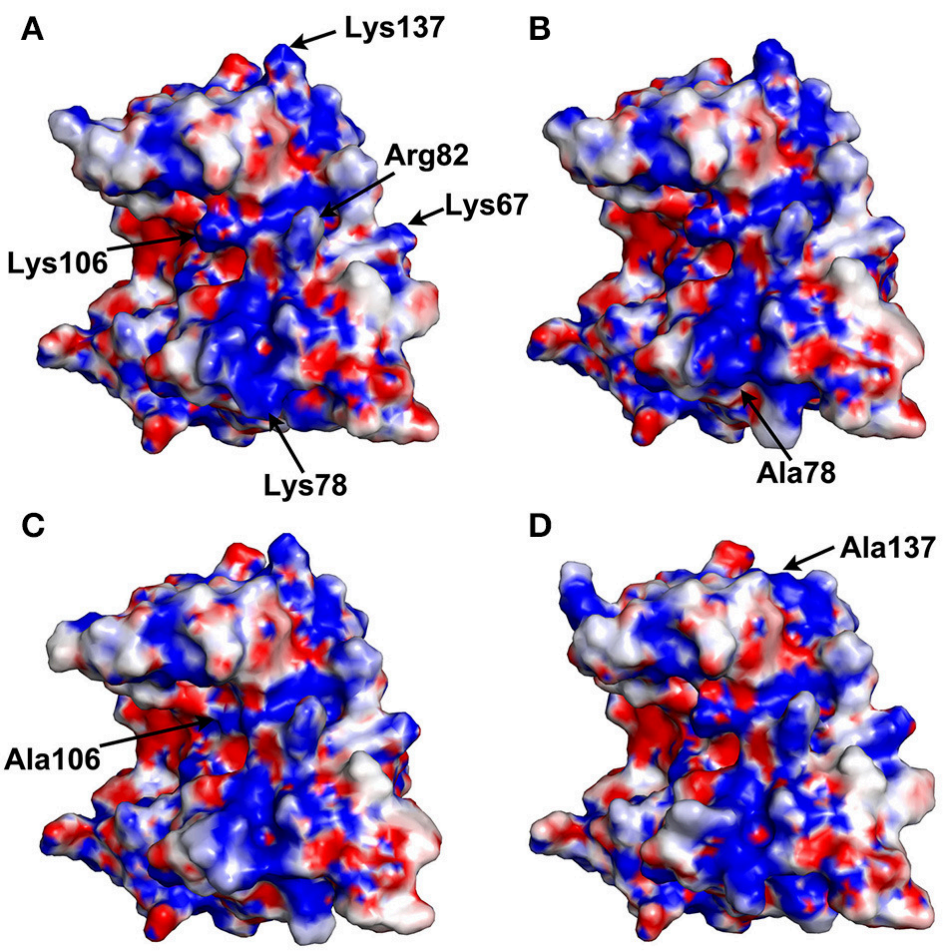
Electrostatic potential surface of thaumatin mutants. The electrostatic potentials of recombinant thaumatin **(A)**, K78A **(B)**, K106A **(C)**, and K137A **(D)** are shown in a surface model from acidic (red) to basic (blue). The residues at positions 67, 78, 82, 106, and 137 are indicated in arrows. The mutated residues are indicated in each panel. Molecular models were generated with *PyMOL*. The structural data of recombinant thaumatin was from PDB entry 3al7 (Masuda et al., 2011a).

The electrostatic potentials of thaumatin mutants as well as recombinant thaumatin were calculated by PDBPQR as a default setting and are indicated in Figure 2. Compared to recombinant thaumatin, the blue color of the mutated site of K78A dramatically changed and the volume of the side-chain was almost lost. These results suggested that the distributions and volume of positive charge from lysine residue were substantially reduced. These tendencies were also found in K137A thaumatin and coincided well with sensory analysis. With K106A thaumatin, the volume of positive charge from lysine residue was reduced but the distribution of charge was relatively small when compared to K78A and K137A. In the three-dimensional structure of thaumatin, Lys106 is located in the cleft center. Compared to Lys78 and Lys137, Lys106 is not relatively exposed to the molecular surface. This subtle difference might result in a slight difference in electrostatic potentials as well as the threshold value of sweetness.

### Interaction thaumatin with T1R2-T1R3 sweet taste receptor

**Figure 3:**
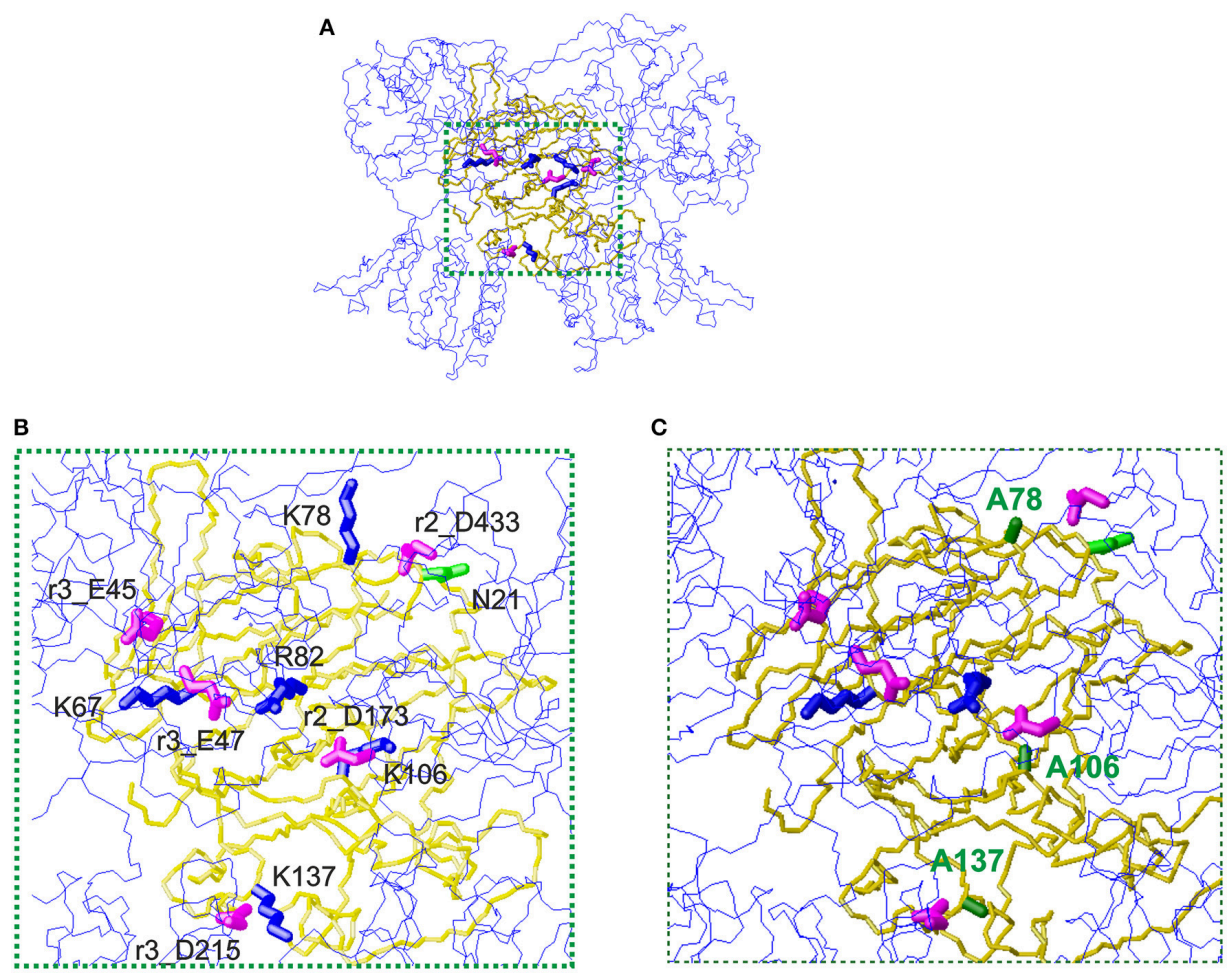
The wedge complex of thaumatin mutants with the T1R2-T1R3 receptor. **(A)** A view of the complex between D21N thaumatin (Masuda et al., 2016). The model of the receptor is shown as a line representation (blue) of the backbone whereas the model of thaumatin is shown as a neon representation of the backbone (gold). The side chains of the key basic residues of thaumatin chosen to optimize the complex are represented as thick blue neons. The corresponding side chains of the acidic residues of the receptor are represented as magenta neons. **(B)** Enlargement of the interaction zone surrounded by green dots in panel **(A)**. This view shows the side of the sweet protein in contact with the receptor. Receptor residues are labeled with the prefix r2_ when belonging to the T1R2 protomer and with r3_ when belonging to the T1R3 protomer respectively. **(C)** Corresponding enlargement of the putative complexes of K78A, K106A and K137A-thaumatin with the T1R2-T1R3 receptor. The alanines are shown with green labels. It is easy to see that three important positive-negative interactions disappear. Models were built with MOLMOL (Koradi et al., 1996).

The first mechanism for the interaction of thaumatin with the sweet receptor was proposed in the framework of a general model of interaction of the three major sweet-tasting proteins (monellin, brazzein, and thaumatin) with the sweet receptor, the so-called wedge model (Temussi, 2002; Morini et al., 2005). In formulating the wedge model, these authors used the GRAMM software (Vasker et al., 1999), since it can be used at low resolution (Temussi, 2002; Morini et al., 2005). Recently, to overcome the lack of resolution of the original approach, more accurate topological models of the receptor with two sweet-tasting proteins, brazzein and monellin have successfully been built by applying a tethered docking procedure in which the use of mutagenesis data and the distribution of charged residues on the interface are explicitly taken into account (Temussi, 2011). By using this procedure, we resorted to mutagenesis data for choosing the most reliable orientation among the complexes originally furnished by GRAMM (Temussi, 2002) to build an accurate model for thaumatin and eventually for its mutants. The models were refined by imposing the consistency of all mutual motions with the mutagenesis data as mentioned before (Temussi, 2011). Since the D21N mutation resulted in the increase of sweetness, we set to investigate this result in the framework of existing models of interaction of thaumatin with sweet receptor (Masuda et al., 2016). The wedge complex between D21N thaumatin is shown in Figure 3A. Enlargement of the putative complexes of D21N-thaumatin and those of K78A, K106A, and K137A-thaumatin with the T1R2-T1R3 sweet receptor are shown in Figures 3B,C, respectively. Two most critical residues (Lys67 and Arg82) in thaumatin are still located in the adjacent to the counterpart residues of sweet receptor (Glu45_T1R3 and Glu47_T1R3 for Lys67, Asp173_T1R2 for Arg82), suggesting that mutations K78A-, K106A-, and K137A-thaumatin, while decreasing local productive interactions with their negative counterparts, were not detrimental to the overall architecture of positive/negative interaction in the interface. As shown in Figure 3B, three lysine residues (Lys78, Lys106, and Lys137) are still tightly interacting with the negatively charged residues on the sweet receptor, that is, NZ of Lys78 is 4.5 Å from CG of Asp433_T1R2, NZ of Lys137 is 5.0 Å from CB of Asp215_T1R3, and NZ of Lys106 interact with CG of Asp173_T1R2, suggesting all these tight interactions reflect a good complementarity. In contrast, three important positive-negative interactions disappear for K78A-, K106A-, and K137A-thaumatin (Figure 3C).

Substantial reductions of sweetness were found in K78A and K137A mutants compared to K106A. As mentioned above, the flexibility of the side-chain for Lys106 is lower than that of for Lys78 and Lys137, and the higher flexible conformations at interaction sites might help complementarily interaction with receptor. In our interaction model, two flexible residues, i.e., Lys78 and Lys137 interacted with the sweet receptor within 5.0 Å. As to Lys106, it was 5.0 Å from CG of Asp173_T1R2, which also interacted with Arg82 in the length of 4.6 Å. Since the interaction to Asp173_T1R2 still is retained, substantial reduction of sweetness might not be induced by K106A.

In summary, this comparative structural study of thaumatin revealed the importance of positively charged patches on interaction sites and the existence of relatively flexible conformations at Lys78 and Lys137. The subtle rearrangements of charge distribution would lead to reduced interaction areas and thus presumably reduce the sweet taste of thaumatin. Understanding the precise structural features as well as charge distributions of thaumatin will provide new insights into the mechanisms of elicitation of sweetness of thaumatin and offer a structural basis for usage in food products and the design of novel sweeteners.

## Author contributions

TM: conceived the research; TM and SK: prepared the crystals; TM, SK, KO, and BM: performed the data collection; TM, SK, MM, and MS: performed data processing and elucidated the structure; TM: wrote the manuscript. All authors reviewed and commented on the results and the manuscript.

### Conflict of interest statement

The authors declare that the research was conducted in the absence of any commercial or financial relationships that could be construed as a potential conflict of interest.

## Supplementary material

Supplementary Figure 1 Superposition of thaumatin mutants on recombinant thaumatin. Superposed K78A (red), K106A (cyan), and K137A (green) on recombinant thaumatin (gray). Each structure is indicated in Cα model. Molecular models were generated with *PyMOL*.

Supplementary Figure 2 E.S.D. analysis of thaumatin mutants. Plot of positional uncertainty versus the thermal parameter for C atoms (black), N atoms (cyan), and O atoms (red). **(A)** K78A, **(B)** K106A, and **(C)** K137A.

## References

[B1] Asherie N. Jakoncic J. Ginsberg C. Greenbaum A. Stojano V. Hrnjez B. J. (2009). Tartrate chirality determines thaumatin crystal habit. Cryst. Growth Des. 9, 4189–4198. 10.1021/cg900465h

[B2] Assadi-Porter F. M. Aceti D. J. Markley J. L. (2000). Sweetness determinant sites of brazzein, a small, heat-stable, sweet-tasting protein. Arch. Biochem. Biophys. 376, 259–265. 10.1006/abbi.2000.1726 10775411

[B3] Assadi-Porter F. M. Maillet E. L. Radek J. T. Quijada J. Markley J. L. Max M. (2010a). Key amino acid residues involved in multi-point binding interactions between brazzein, a sweet protein, and the T1R2-T1R3 human sweet receptor. J. Mol. Biol. 398, 584–599. 10.1016/j.jmb.2010.03.017 20302879 PMC2879441

[B4] Assadi-Porter F. M. Tonelli M. Maillet E. L. Markley J. L. Max M. (2010b). Interactions between the human sweet-sensing T1R2-T1R3 receptor and sweeteners detected by saturation transfer difference NMR spectroscopy. Biochim. Biophys. Acta 1798, 82–86. 10.1016/j.bbamem.2009.07.021 19664591 PMC2814067

[B5] Blake C. C. Johnson L. N. Mair G. A. North A. C. Phillips D. C. Sarma V. R. (1967). Crystallographic studies of the activity of hen egg-white lysozyme. Proc. R. Lond. Ser. B Biol. Sci. 167, 378–388. 10.1098/rspb.1967.0035 4382801

[B6] Brünger A. T. (1992). Free R value: a novel statistical quantity for assessing the accuracy of crystal structures. Nature 355, 472–475. 10.1038/355472a0 18481394

[B7] Caldwell J. E. Abildgaard F. Dzakula Z. Ming D. Hellekant G. Markley J. L. (1998). Solution structure of the thermostable sweet-tasting protein brazzein. Nat. Struct. Biol. 5, 427–431. 10.1038/nsb0698-427 9628478

[B8] Chandrashekar J. Hoon M. A. Ryba N. J. Zuker C. S. (2006). The receptors and cells for mammalian taste. Nature 444, 288–294. 10.1038/nature05401 17108952

[B9] Clemmensen C. Smajilovic S. Wellendorph P. Bräuner-Osborne H. (2014). The GPCR, class C, group 6, subtype A (GPRC6A) receptor: from cloning to physiological function. Br. J. Pharmacol. 171, 1129–1141. 10.1111/bph.12365 24032653 PMC3952793

[B10] Cornilescu C. C. Cornilescu G. Rao H. Porter S. F. Tonelli M. DeRider M. L.. (2013). Temperature-dependent conformational change affecting Tyr11 and sweetness loops of brazzein. Proteins 81, 919–925. 10.1002/prot.24259 23349025 PMC3982881

[B11] Cruickshank D. W. J. (1999). Remarks about protein structure precision. Acta Crystallogr. D Biol. Crystallogr. 55, 583–601. 10.1107/S0907444998012645 10089455

[B12] DeLano W. L. (2002). The PyMOL Molecular Graphics System. San Carlos, CA: DeLano Scientific

[B13] Dittli S. M. Rao H. Tonelli M. Quijada J. Markley J. L. Max M.. (2011). Structural role of the terminal disulfide bond in the sweetness of brazzein. Chem. Senses 36, 821–830. 10.1093/chemse/bjr057 21765060 PMC3195789

[B14] Dolinsky T. J. Nielsen J. E. McCammon J. A. Baker N. A. (2004). PDB2PQR: an automated pipeline for the setup, execution, and analysis of Poisson-Boltzmann electrostatics calculations. Nucleic Acids Res. 32, W665–W667. 10.1093/nar/gkh381 15215472 PMC441519

[B15] Emsley P. Cowtan K. (2004). Coot: model-building tools for molecular graphics. Acta Crystallogr. D Biol. Crystallogr. 60, 2126–2132. 10.1107/S0907444904019158 15572765

[B16] Esposito V. Gallucci R. Picone D. Saviano G. Tancredi T. Temussi P. A. (2006). The importance of electrostatic potential in the interaction of sweet proteins with the sweet taste receptor. J. Mol. Biol. 360, 448–456. 10.1016/j.jmb.2006.05.020 16764888

[B17] Etheridge K. (1994). The sales and marketing of talin, in Thaumatin, eds Witty M. Higginbotham J. D. (Boca Raton, FL: CRC Press), 47–59.

[B18] Ghanavatian P. Khalifeh K. Jafarian V. (2016). Structural features and activity of Brazzein and its mutants upon substitution of a surfaced exposed alanine. Biochimie 131, 20–28. 10.1016/j.biochi.2016.09.006 27616457

[B19] Gruene T. Hahn H. W. Luebben A. V. Meilleur F. Sheldrick G. M. (2013). Refinement of macromolecular structures against neutron data with SHELXL2013. J. Appl. Crystallogr. 47, 462–466. 10.1107/S1600576713027659 24587788 PMC3937812

[B20] Hobbs J. R. Munger S. D. Conn G. L. (2007). Monellin (MNEI) at 1.15 Å resolution. Acta Crystallogr. F Biol. Crystallogr. 63, 162–167. 10.1107/S1744309107005271 17329805 PMC2330190

[B21] Ide N. Masuda T. Kitabatake N. (2007). Effects of pre- and pro-sequence of thaumatin on the secretion by *Pichia pastoris*. Biochem. Biophys. Res. Commun. 363, 708–714. 10.1016/j.bbrc.2007.09.021 17897626

[B22] Ide N. Sato E. Ohta K. Masuda T. Kitabatake N. (2009). Interactions of the sweet-tasting proteins thaumatin and lysozyme with the human sweet-taste receptor. J. Agric. Food Chem. 57, 5884–5890. 10.1021/jf803956f 19489607

[B23] Jiang P. Ji Q. Liu Z. Snyder L. A. Benard L. M. Margolskee R. F.. (2004). The cysteine-rich region of T1R3 determines responses to intensely sweet proteins. J. Biol. Chem. 279, 45068–45075. 10.1074/jbc.M406779200 15299024

[B24] Jin Z. Danilova V. Assadi-Porter F. M. Aceti D. J. Markley J. L. Hellekant G. (2003). Critical regions for the sweetness of brazzein. FEBS Lett. 544, 33–37. 10.1016/S0014-5793(03)00383-1 12782286

[B25] Kaneko R. Kitabatake N. (2001). Structure-sweetness relationship in thaumatin: importance of lysine residues. Chem. Senses 26, 167–177. 10.1093/chemse/26.2.167 11238247

[B26] Kim S. -H. Weickmann J. L. (1994). Crystal structure of thaumatin I and its correlation to biochemical and mutational studies, in Thaumatin, eds Witty M. Higginbotham J. D. (Boca Raton, FL: CRC Press), 135–149.

[B27] Ko T. P. Day J. Greenwood A. McPherson A. (1994). Structures of three crystal forms of the sweet protein thaumatin. Acta Crystallogr. D Biol. Crystallogr. 50, 813–825. 10.1107/S0907444994005512 15299348

[B28] Kohmura M. Nio N. Ariyoshi Y. (1992). Highly probable active site of the sweet protein monellin. Biosci. Biotechnol. Biochem. 56, 1937–1942. 10.1271/bbb.56.1937 1369093

[B29] Koizumi A. Nakajima K. Asakura T. Morita Y. Ito K. Shmizu-Ibuka A.. (2007). Taste-modifying sweet protein, neoculin, is received at human T1R3 amino terminal domain. Biochem. Biophys. Res. Commun. 358, 585–589. 10.1016/j.bbrc.2007.04.171 17499612

[B30] Koizumi T. Terada T. Nakajima K. Kojima M. Koshiba S. Matsumura Y.. (2015). Identification of key neoculin residues responsible for the binding and activation of the sweet taste receptor. Sci. Rep. 5:12947. 10.1038/srep12947 26263392 PMC4542694

[B31] Koradi R. Billeter M. Wüthrich K. (1996). MOLMOL: a program for display and analysis of macromolecular structures. J. Mol. Graph. 14, 29–32. 10.1016/0263-7855(96)00009-4 8744573

[B32] Kunishima N. Shimada Y. Tsuji Y. Sato T. Yamamoto M. Kumasaka T.. (2000). Structural basis of glutamate recognition by a dimeric metabotropic glutamate receptor. Nature 407, 971–977. 10.1038/35039564 11069170

[B33] Kurimoto E. Suzuki M. Amemiya E. Yamaguchi Y. Nirasawa S. Shimba N.. (2007). Curculin exhibits sweet-tasting and taste-modifying activities through its distinct molecular surfaces. J. Biol. Chem. 282, 33252–33256. 10.1074/jbc.C700174200 17895249

[B34] Laskowski R. A. MacArthur M. W. Moss D. S. Thornton J. M. (1993). PROCHECK: a program to check the stereochemical quality of protein structures. J. Appl. Cryst. 26, 283–291. 10.1107/S0021889892009944

[B35] Lee J. W. Cha J. E. Jo H. J. Kong K. H. (2013). Multiple mutations of the critical amino acid residues for the sweetness of the sweet-tasting protein, brazzein. Food Chem. 138, 1370–1373. 10.1016/j.foodchem.2012.10.140 23411256

[B36] Lee S. Y. Lee J. H. Chang H. J. Cho J. M. Jung J. W. Lee W. (1999). Solution structure of a sweet protein single-chain monellin determined by nuclear magnetic resonance and dynamical simulated annealing calculations. Biochemistry 38, 2340–2346. 10.1021/bi9822731 10029527

[B37] Leone S. Pica A. Merlino A. Sannino F. Temussi P. A. Picone D. (2016). Sweeter and stronger: enhancing sweetness and stability of the single chain monellin MNEI through molecular design. Sci. Rep. 6:34045. 10.1038/srep34045 27658853 PMC5034325

[B38] Li D. F. Jiang P. Zhu D. Y. Hu Y. Max M. Wang D. C. (2008). Crystal structure of Mabinlin II: a novel structural type of sweet proteins and the main structural basis for its sweetness. J. Struct. Biol. 162, 50–62. 10.1016/j.jsb.2007.12.007 18308584

[B39] Li X. Staszewski L. Xu H. Durick K. Zoller M. Adler E. (2002). Human receptors for sweet and umami taste. Proc. Natl. Acad. Sci. U.S.A. 99, 4692–4696. 10.1073/pnas.072090199 11917125 PMC123709

[B40] Lim J. K. Jang J. C. Kong J. N. Kim M. C. Kong K. H. (2016). Importance of Glu53 in the C-terminal region of brazzein, a sweet-tasting protein. J. Sci. Food Agric. 96, 3202–3206. 10.1002/jsfa.7501 26478244

[B41] Liu B. Ha M. Meng X. Y. Khaleduzzaman M. Zhang Z. Li X.. (2012). Functional characterization of the heterodimeric sweet taste receptor T1R2 and T1R3 from a New World monkey species (squirrel monkey) and its response to sweet-tasting proteins. Biochem. Biophys. Res. Commun. 427, 431–437. 10.1016/j.bbrc.2012.09.083 23000410 PMC3479362

[B42] Liu Q. Li L. Yang L. Liu T. Cai C. Liu B. (2016). Modification of the sweetness and stability of sweet-tasting protein monellin by gene mutation and protein engineering. Biomed. Res. Int. 2016:3647173. 10.1155/2016/3647173 26881217 PMC4736911

[B43] Liu X. Maeda S. Hu Z. Aiuchi T. Nakaya K. Kurihara Y. (1993). Purification, complete amino acid sequence and structure characterization of the heat stable sweet protein, mabinlin II. Eur. J. Biochem. 211, 281–287. 10.1111/j.1432-1033.1993.tb19896.x 8425538

[B44] Lovell S. C. Davis I. W. Arendall W. B. III. de Bakker P. I. W. Word J. M. Prisant M. G. (2003). Structure validation by Cα geometry: ϕ, ψ and Cβ deviation. Proteins 50, 437–450. 10.1002/prot.10286 12557186

[B45] Maehashi K. Udaka S. (1998). Sweetness of lysozyme. Biosci. Biotechnol. Biochem. 53, 605–606. 10.1271/bbb.62.605 9571795

[B46] Masuda T. (2018). Sweet-tasting protein thaumatin: physical and chemical properties, in Sweeteners, Part of the Series Reference Series in Phytochemistry, eds Merillon J. -M. Ramawat K. G. (Cham: Springer), 493–523. 10.1007/978-3-319-27027-2_10

[B47] Masuda T. Ide N. Kitabatake N. (2005a). Effects of chemical modification of lysine residues on the sweetness of lysozyme. Chem. Senses 30, 253–264. 10.1093/chemse/bji021 15741597

[B48] Masuda T. Ide N. Kitabatake N. (2005b). Structure-sweetness relationship in egg white lysozyme: role of lysine and arginine residues on the elicitation of lysozyme sweetness. Chem. Senses 30, 667–681. 10.1093/chemse/bji060 16162643

[B49] Masuda T. Ide N. Ohta K. Kitabatake N. (2010). High-yield secretion of the recombinant sweet-tasting protein thaumatin I. Food Sci. Technol. Res. 16, 585–592. 10.3136/fstr.16.585

[B50] Masuda T. Mikami B. Tani F. (2014). Atomic structure of recombinant thaumatin II reveals flexible conformations in two residues critical for sweetness and three consecutive glycine residues. Biochimie 106, 33–38. 10.1016/j.biochi.2014.07.016 25066915

[B51] Masuda T. Ohta K. Mikami B. Kitabatake N. (2011a). High-resolution structure of the recombinant sweet-tasting protein thaumatin I. Acta Crystallogr. Sect. F Struct. Biol. Cryst. Commun. 67, 652–658. 10.1107/S174430911101373X 21636903 PMC3107134

[B52] Masuda T. Ohta K. Ojiro N. Murata K. Mikami B. Tani F.. (2016). A hypersweet protein: removal of the specific negative charge at Asp21 enhances thaumatin sweetness. Sci. Rep. 6:20255. 10.1038/srep20255 26837600 PMC4738316

[B53] Masuda T. Ohta K. Tani F. Mikami B. Kitabatake N. (2011b). Crystal structure of the sweet-tasting protein thaumatin II at 1.27 Å. Biochem. Biophys. Res. Commun. 410, 457–460. 10.1016/j.bbrc.2011.05.158 21672520

[B54] Masuda T. Taguchi W. Sano A. Ohta K. Kitabatake N. Tani F. (2013). Five amino acid residues in cysteine-rich domain of human T1R3 were involved in the response for sweet-tasting protein, thaumatin. Biochimie 95, 1502–1505. 10.1016/j.biochi.2013.01.010 23370115

[B55] Masuda T. Ueno Y. Kitabatake N. (2001). Sweetness and enzymatic activity of lysozyme. J. Agric. Food Chem. 49, 4937–4941. 10.1021/jf010404q 11600047

[B56] Ming D. Hellekant G. (1994). Brazzein, a new high-potency thermostable sweet protein from Pentadiplandra brazzeana B. FEBS Lett. 355, 106–108. 10.1016/0014-5793(94)01184-2 7957951

[B57] Morini G. Bassoli A. Temussi P. A. (2005). From small sweeteners to sweet proteins: anatomy of the binding sites of the human T1R2_T1R3 receptor. J. Med. Chem. 48, 5520–5529. 10.1021/jm0503345 16107151

[B58] Moris J. A. Cagan R. H. (1972). Purification of monellin, the sweet principal for *Dioscoreophyllum cumminsii*. Biochim. Biophys. Acta. 261, 114–122. 10.1016/0304-4165(72)90320-0 5012458

[B59] Nabors L. O'. Gelardi R. C. (1991). Alternative Sweeteners. New York, NY: Marcel Dekker.

[B60] Nagata K. Hongo N. Kameda Y. Yamamura A. Sasaki H. Lee W. C.. (2013). The structure of brazzein, a sweet-tasting protein from the wild African plant *Pentadiplandra brazzeana*. Acta Crystallogr. D Biol. Crystallogr. 69, 642–647. 10.1107/S0907444913001005 23519673

[B61] Nakajima K. Morita Y. Koizumi A. Asakura T. Terada T. Ito K.. (2008). Acid-induced sweetness of neoculin is ascribed to its pH-dependent agonistic-antagonistic interaction with human sweet taste receptor. FASEB J. 22, 2323–2330. 10.1096/fj.07-100289 18263698

[B62] Nakajima K. Yokoyama K. Koizumi T. Koizumi A. Asakura T. Terada T.. (2011). Identification and modulation of the key amino acid residue responsible for the pH sensitivity of neoculin, a taste-modifying protein. PLoS ONE 6:e19448. 10.1371/journal.pone.0019448 21559382 PMC3084864

[B63] Nelson G. Hoon M. A. Chandrashekar J. Zhang Y. Ryba N. J. Zuker C. S. (2001). Mammalian sweet taste receptors. Cell 106, 381–390. 10.1016/S0092-8674(01)00451-2 11509186

[B64] Ogata C. M. Gordon P. F. de Vos A. M. Kim S. H. (1992). Crystal structure of a sweet tasting protein thaumatin I, at 1.65 Å resolution. J. Mol. Biol. 228, 893–908. 10.1016/0022-2836(92)90873-I 1469722

[B65] Ohta K. Masuda T. Ide N. Kitabatake N. (2008). Critical molecular regions for elicitation of the sweetness of the sweet-tasting protein thaumatin I. FEBS J. 275, 3644–3652. 10.1111/j.1742-4658.2008.06509.x 18544096

[B66] Ohta K. Masuda T. Tani F. Kitabatake N. (2011a). The cysteine-rich domain of human T1R3 is necessary for the interaction between human T1R2-T1R3 sweet receptors and a sweet-tasting protein, thaumatin. Biochem. Biophys. Res. Commun. 406, 435–438. 10.1016/j.bbrc.2011.02.063 21329673

[B67] Ohta K. Masuda T. Tani F. Kitabatake N. (2011b). Introduction of a negative charge at Arg82 in thaumatin abolished responses to human T1R2-T1R3 sweet receptors. Biochem. Biophys. Res. Commun. 413, 41–45. 10.1016/j.bbrc.2011.08.033 21867681

[B68] Otwinowski Z. Minor W. (1997). Processing of X-ray crystallographic data collected in oscillation mode. Methods Enzymol. 276, 307–326. 10.1016/S0076-6879(97)76066-X 27754618

[B69] Phillips D. C. (1967). The hen egg white lysozyme molecule. Proc. Natl. Acad. Sci. U.S.A. 57, 484–495. 10.1098/rspb.1967.0034 4382800

[B70] Rega M. F. Di Monaco R. Leone S. Donnarumma F. Spadaccini R. Cavella S.. (2015). Design of sweet protein based sweeteners: hints from structure-function relationships. Food Chem. 173, 1179–1186. 10.1016/j.foodchem.2014.10.151 25466141

[B71] Shamil S. Beynon R. J. (1990). A structure-activity study of thaumatin using pyridoxal 5'-phosphate (PLP) as a prove. Chem. Senses 15, 457–469. 10.1093/chemse/15.4.457

[B72] Sheldrick M. Schneider T. R. (1997). SHELXL: high-resolution refinement. Methods Enzymol. 277, 319–343. 10.1016/S0076-6879(97)77018-6 18488315

[B73] Shimizu-Ibuka A. Morita Y. Terada T. Asakura T. Nakajima K. Iwata S.. (2006). Crystal structure of neoculin: insights into its sweetness and taste-modifying activity. J. Mol. Biol. 359, 148–158. 10.1016/j.jmb.2006.03.030 16616933

[B74] Shirasuka Y. Nakajima K. Asakura T. Yamashita H. Yamamoto A. Hata S.. (2004). Neoculin as a new taste-modifying protein occurring in the fruit of Curculigo latifolia. Biosci. Biotechnol. Biochem. 68, 1403–1407. 10.1271/bbb.68.1403 15215616

[B75] Singarapu K. K. Tonelli M. Markley J. L. Assadi-Porter F. M. (2016). Structure-function relationships of brazzein variants with altered interactions with the human sweet taste receptor. Protein Sci. 25, 711–719. 10.1002/pro.2870 26701738 PMC4815422

[B76] Slootstra J. W. De Geus P. Haas H. Verrips C. T. Meloen R. H. (1995). Possible active site of the sweet-tasting protein thaumatin. Chem. Senses 20, 535–543. 10.1093/chemse/20.5.535 8564428

[B77] Smith L. J. Sutcliffe M. J. Redfield C. Dobson C. M. (1993). Structure of hen lysozyme in solution. J. Mol. Biol. 229, 930–994. 10.1006/jmbi.1993.1097 8445657

[B78] Somoza J. R. Cho J. M. Kim S. H. (1995). The taste-active regions of monellin, a potently sweet protein. Chem. Senses 20, 61–68. 10.1093/chemse/20.1.61 7796059

[B79] Somoza J. R. Jiang F. Tong L. Kang C. H. Cho J. M. Kim S. H. (1993). Two crystal structures of a potently sweet protein. Natural monellin at 2.75 Å resolution and single-chain monellin at 1.7 Å resolution. J. Mol. Biol. 234, 390–404. 10.1006/jmbi.1993.1594 8230222

[B80] Spadaccini R. Crescenzi O. Tancredi T. De Casamassimi N. Saviano G. Scognamiglio R.. (2001). Solution structure of a sweet protein: NMR study of MNEI, a single chain monellin. J. Mol. Biol. 305, 505–514. 10.1006/jmbi.2000.4304 11152608

[B81] Suzuki M. Kurimoto E. Nirasawa S. Masuda Y. Hori K. Kurihara Y.. (2004). Recombinant curculin heterodimer exhibits taste-modifying and sweet-tasting activities. FEBS Lett. 573, 135–138. 10.1016/j.febslet.2004.07.073 15327988

[B82] Tancredi T. Pastore A. Salvadori S. Esposito V. Temussi P. A. (2004). Interaction of sweet proteins with their receptor. A conformational study of peptides corresponding to loops of brazzein, monellin and thaumatin. Eur. J. Biochem. 271, 2231–2240. 10.1111/j.1432-1033.2004.04154.x 15153113

[B83] Templeton C. M. Ostovar pour S. Hobbs J. R. Blanch E. W. Munger S. D. Conn G. L. (2011). Reduced sweetness of a monellin (MNEI) mutant results from increased protein flexibility and disruption of a distant poly-(L-proline) II helix. Chem. Senses 36, 425–434. 10.1093/chemse/bjr007 21343241 PMC3094690

[B84] Temussi P. A. (2002). Why are sweet proteins sweet? Interaction of brazzein, monellin and thaumatin with the T1R2-T1R3 receptor. FEBS Lett. 526, 1–4. 10.1016/S0014-5793(02)03155-1 12208493

[B85] Temussi P. A. (2006). Natural sweet macromoleculars: how sweet protein work. Cell Mol. Life Sci. 63, 1876–1888. 10.1007/s00018-006-6077-8 16810455 PMC11136200

[B86] Temussi P. A. (2011). Determinants of sweetness in proteins: a topological approach. J. Mol. Recognit. 24, 1033–1042. 10.1002/jmr.1152 22038810

[B87] Ueno G. Kanda H. Hirose R. Ida K. Kumasaka T. Yamamoto M. (2006). RIKEN structural genomics beamlines at the SPring-8; high throughput protein crystallography with automated beamline operation. J. Struct. Funct. Genomics 7, 15–22. 10.1007/s10969-005-9005-5 16645781

[B88] van der Wel H. (1972). Isolation and characterization of the sweet principal for Dioscoreophyllum cumminsii (Stapf) Diels. FEBS Lett. 21, 88–90. 10.1016/0014-5793(72)80170-4 11946482

[B89] van der Wel H. (1994). Structure-activity relationship in the thaumatin molecule, in Thaumatin, eds Witty M. Higginbotham J. D. (Boca Raton, FL: CRC Press), 115–122.

[B90] van der Wel H. Bel W. J. (1976). Effect of acetylation and methylation on the sweetness intensity of thaumatin I. Chem. Senses 2, 211–218. 10.1093/chemse/2.2.211

[B91] van der Wel H. Loeve K. (1972). Isolation and characterization of thaumatin I and II, the sweet-tasting proteins from *Thaumatococcus daniellii* Benth. Eur. J. Biochem. 31, 221–225. 10.1111/j.1432-1033.1972.tb02522.x 4647176

[B92] Vasker I. A. Matar O. G. Lam C. F. (1999). A systematic study of low-resolution recognition in protein–protein complexes. Proc. Natl. Acad. Sci. U.S.A. 96, 8477–8482. 10.1073/pnas.96.15.8477 10411900 PMC17541

[B93] Walters D. E. Cragin T. Jin Z. Rumbley J. N. Hellekant G. (2009). Design and evaluation of new analogs of the sweet protein brazzein. Chem. Senses 34, 679–683. 10.1093/chemse/bjp048 19696120 PMC2745351

[B94] Walters D. E. Hellekant G. (2006). Interactions of the sweet protein brazzein with the sweet taste receptor. J. Agric. Food Chem. 54, 10129–10133. 10.1021/jf062359y 17177550 PMC2527743

[B95] Winn M. D. Ballard C. C. Cowtan K. D. Dodson E. J. Emsley P. Evans P. R.. (2011). Overview of the CCP4 suite and current developments. Acta Crystallogr. D Biol. Crystallogr. 67, 235–242. 10.1107/S0907444910045749 21460441 PMC3069738

[B96] Xu H. Staszewski L. Tang H. Adler E. Zoller M. Li X. (2004). Different functional roles of T1R subunits in the heteromeric taste receptors. Proc. Natl. Acad. Sci. U.S.A. 101, 14258–14263. 10.1073/pnas.0404384101 15353592 PMC521102

[B97] Xue W. F. Szczepankiewicz O. Thulin E. Linse S. Carey J. (2009). Role of protein surface charge in monellin sweetness. Biochim. Biophys. Acta 1794, 410–420. 10.1016/j.bbapap.2008.11.008 19100868

[B98] Yamashita H. Theerasilp S. Aiuchi T. Nakaya K. Nakamura Y. Kurihara Y. (1990). Purification and complete amino acid sequence of a new type of sweet protein with taste-modifying activity, curculin. J. Biol. Chem. 265, 15770–15775. 2394746

