# Supplementary figures and images for: Positive Charges on the Surface of Thaumatin Are Crucial for the Multi-Point Interaction with the Sweet Receptor
Source: Front Mol Biosci. 2018 Feb 13;5:10. doi: 10.3389/fmolb.2018.00010 (PMC5816810; doi:10.3389/fmolb.2018.00010)

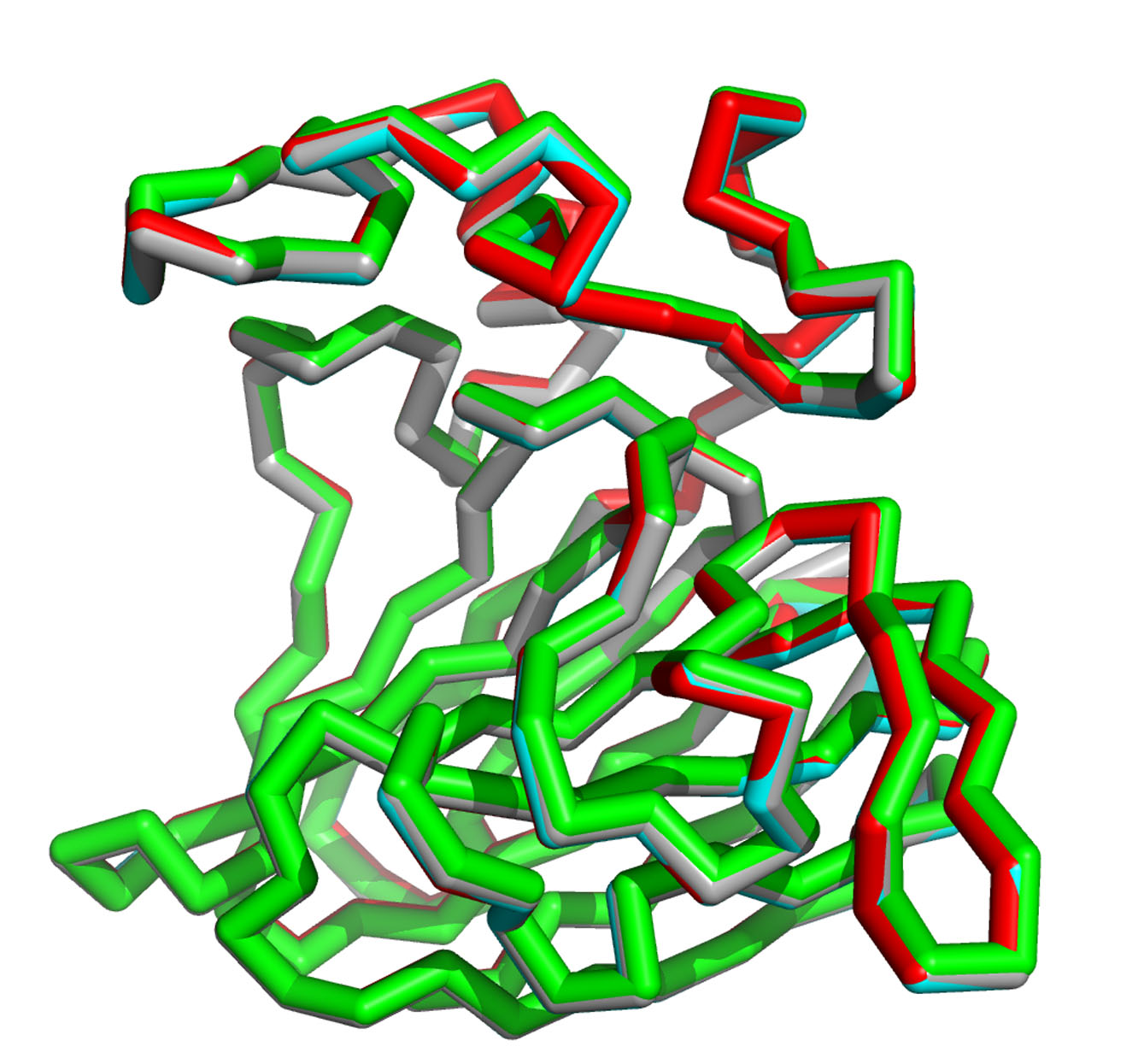

Supplement: Supplementary Figure 1 — Superposition of thaumatin mutants on recombinant thaumatin. Superposed K78A (red), K106A (cyan), and K137A (green) on recombinant thaumatin (gray). Each structure is indicated in Cα model. Molecular models were generated with PyMOL. [file Image1.JPEG]

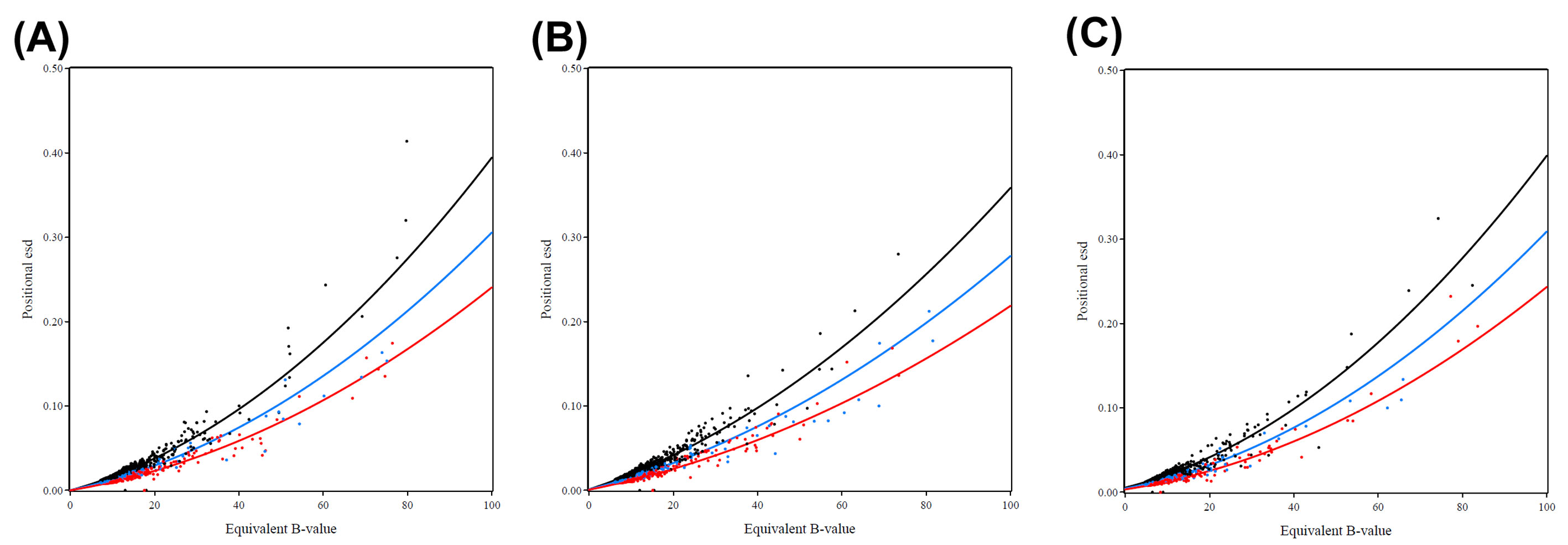

Supplement: Supplementary Figure 2 — E.S.D. analysis of thaumatin mutants. Plot of positional uncertainty versus the thermal parameter for C atoms (black), N atoms (cyan), and O atoms (red). (A) K78A, (B) K106A, and (C) K137A. [file Image2.JPEG]
